# Racism and health among urban Aboriginal young people

**DOI:** 10.1186/1471-2458-11-568

**Published:** 2011-07-15

**Authors:** Naomi Priest, Yin Paradies, Paul Stewart, Joanne Luke

**Affiliations:** 1 McCaughey Centre and Onemda VicHealth Koori Health Unit, Melbourne School of Population Health, University of Melbourne, Melbourne, Australia; 2 Onemda VicHealth Koori Health Unit, Melbourne School of Population Health, University of Melbourne, Melbourne, Australia; 3 Victorian Aboriginal Health Service, Melbourne, Australia

## Abstract

**Background:**

Racism has been identified as an important determinant of health but few studies have explored associations between racism and health outcomes for Australian Aboriginal young people in urban areas.

**Methods:**

Cross sectional data from participants aged 12-26 years in Wave 1 of the Victorian Aboriginal Health Service's Young People's Project were included in hierarchical logistic regression models. Overall mental health, depression and general health were all considered as outcomes with self-reported racism as the exposure, adjusting for a range of relevant confounders.

**Results:**

Racism was reported by a high proportion (52.3%) of participants in this study. Self-reported racism was significantly associated with poor overall mental health (OR 2.67, 95% CI 1.25-5.70, p = 0.01) and poor general health (OR 2.17, 95% CI 1.03-4.57, p = 0.04), and marginally associated with increased depression (OR 2.0; 95% CI 0.97-4.09, p = 0.06) in the multivariate models. Number of worries and number of friends were both found to be effect modifiers for the association between self-reported racism and overall mental health. Getting angry at racist remarks was found to mediate the relationship between self-reported racism and general health.

**Conclusions:**

This study highlights the need to acknowledge and address racism as an important determinant of health and wellbeing for Aboriginal young people in urban areas of Australia.

## Background

Throughout the world, there is increasingly recognition that racism is an important determinant of health for indigenous populations and other minority groups [1,2]. Racism can be expressed as beliefs, stereotypes, prejudices or discrimination and can range from open threats and insults to being deeply embedded in social systems and structures [3]. Thus racism can be defined as phenomena that results in avoidable and unfair inequalities in power, resources and opportunities across racial or ethnic groups [3].

The substantial inequalities in health and wellbeing experienced by Aboriginal and Torres Strait Islander peoples (including children and young people) is well established, and addressing these inequalities are high on the Australian national agenda [4]. Historical and contemporary racism, colonisation and oppression are identified as key determinants of these inequalities [5] and addressing racism as a determinant of Aboriginal and Torres Strait Islander health has also been recognised at a national level [5-9].

The study of racism as a determinant of health is an emerging field of research. Increasingly, international studies are showing strong associations between self-reported racism and poor adult health outcomes across a range of minority groups in developed countries, after adjusting for confounders and within longitudinal and cross sectional studies [2,10,11]. Systematic reviews in the area identify links between self-reported racism and poor mental health (including depression, anxiety, psychological distress); poor physical health (including hypertension, cardiovascular reactivity, chronic health conditions); and increased substance use [2,10,11].

Despite this, little research specifically investigates the impact of racism on the health and wellbeing of children and young people [2,10,12]. Research that does exist predominantly focuses on African American adolescents in the United States[12]. These studies have shown strong associations between self-reported racism and poor mental health outcomes, indicators of metabolic and cardiovascular disease, and substance use [12,13]. The applicability of these findings to Australian young people, including those from Aboriginal and Torres Strait Islander backgrounds, requires further investigation.

In Australia, the prevalence of racism is documented by recent surveys, [14,15] including racism experienced by Australian young people [16]. However, there is a paucity of research on the relationship between racism and the health and wellbeing of Australian children and young people (including those from Aboriginal and Torres Strait Islander backgrounds) [17,18]. One large population study reported associations between self reported racism and substance use, emotional and behavioural difficulties, and suicide risk for Aboriginal young people aged 12-17 years in Western Australia[13].While this finding is consistent with the few studies exploring racism and health among adult Aboriginal Australians, [5,18-21] more research is needed to understand the impact of racism on the health of Aboriginal children and young people from a diverse range of age groups and geographical locations.

In Victoria, Aboriginal and Torres Strait Islander people represent approximately 0.6% of the total state population, the lowest proportion of any Australian state or territory [22]. With approximately half of the Aboriginal and Torres Strait Islander population living in Melbourne, the state's capital city, Melbourne's Aboriginal and Torres Strait Islander population can be considered particularly vulnerable to racism and challenges of invisibility [23]. The term "Koori" is used by Aboriginal people from Victoria, parts of New South Wales and Tasmania to describe themselves and is also used in this study. This study aimed to explore associations between self-reported racism and health outcomes for Aboriginal young people living in Melbourne, Victoria.

## Methods

### Study setting and design

The Victorian Aboriginal Health Service (VAHS) Young People's Project (YPP) was a study conducted in Melbourne, Australia with Wave 1 in 1997/8 and Wave 2 in 2001. The YPP was one of the first research studies to be initiated and conducted by an Aboriginal community controlled health service in Australia. The approach and methods have been described elsewhere [24]. Participants were recruited through the community controlled health service and via community networks with data collected by peer interviewers at community meeting places [24]. This study utilises cross-sectional survey data from Wave 1 of the YPP in which Aboriginal young people aged 12-26 years from Melbourne completed a health questionnaire and participated in health checks during 1997/98. Wave 2 data were not used due to high loss to follow up. Approval for this analysis was obtained from the VAHS research sub-committee and the VAHS Management Board. Original study protocols were approved by the VAHS Institutional Ethics Committee and the VAHS Management Board.

### Health outcomes: Depression, overall mental health and general health

Participants completed items on mental health and depression adapted from review of the Indian Youth Resiliency Impact Study [25], an Indigenous Health Program [26], and the Gatehouse Survey [27]. Participants were asked to respond yes/no to ten questions on depression that correspond to the nine item Personal Health Questionnaire (PHQ-9), a valid and reliable measure of depression severity [28]. In this study, one of the PHQ-9 items was split into two parts thus there were ten depression items (α = 0.83 in this study). In the last two weeks have you had: trouble falling asleep or sleeping too much; feeling tired or having little energy; poor appetite or overeating?; little interest or pleasure in life; feeling down, depressed or hopeless; feeling bad about yourself or that you are a failure, or have let yourself or your family down; trouble concentrating on things; so fidgety or restless that you have been moving around a lot more than usual; moving or speaking so slowly that other people could notice; have you had thoughts you would be better off dead. Responses to each of these items were summed to form a total depression score that was then dichotomised at its median and coded as a binary variable ("no" and "yes") for analysis.

Participants also responded to additional items related to general mental health: Are you happy/satisfied with life?; Is there at least one thing you are good at?; Do you like yourself?; Do you feel anxious?; Do you find you do things over and over again?; do you suffer from aches, pains or sickness because of stress or anxiety?; Do you feel angry?; Have you tried to kill yourself? An overall mental health score was formed by summing responses to these additional questions together with those from the depression items (α = 0.86 in this study). This overall mental health score was then dichotomised at its median and coded as a binary variable ("good" and "poor") for analysis.

Participants were asked: 'How is your health in general?' coded in two groups ("poor/fair" and "good/excellent") for analysis.

### Primary explanatory variables: Self-reported racism

Participants were asked: "Do you feel discriminated against because you are Koori?" and were asked to select one of five responses: not at all, a little, some, quite a bit or a lot. These were coded into two groups for analysis ("not at all" and "a little/some/quite a bit/a lot").

### Secondary explanatory variables

A range of variables describing socio-demographics, life stressors, friends, school, emotions and feelings, and cultural values were included. Participants reported age (years) coded in two groups for analysis (12-17 and 18-26) consistent with 18 years as the legal age of adulthood in Australia; current level of education coded in three groups for analysis (primary/high school, TAFE/University and not a student); and present money situation (plenty of money for what I need, I get by with what I get and not having enough money causes me problems).

Life stressors reported were food security (is getting enough food a problem for you?; never, some days/often) and housing stability (are you looking for somewhere to live at present?; no, yes/unsure). Participants were also asked if they worry about common life stressors (response set: not at all, some and a lot). Stressors were: a close friend might die; your family might not have enough money to buy food; your family might split up; a parent may die; there is drinking or drug use in your home; about violence in your home; about not having someone to take care of you, about not being able to wear the latest brand name clothes/shoes; about getting into fights; about dying soon; about getting AIDS; about gambling in your home). A variable summing responses to twelve individual worries was created and dichotomised at the median for analysis.

Participants were asked about their friends: Do you have no friends, a few friends or a lot of friends?; When you have a problem what do you do to feel better? (talk to friends, yes/no); Participants were asked if they feel safe at school (yes, sometimes/never); have you ever been suspended or expelled from school? (not at all, once/more than once). Emotions and feelings reported were: Do you feel lonely? (never, sometimes/often/always); and do you get angry at racist remarks? (yes/no). Participants were also asked: Are Koori values important to you? (not at all, somewhat/very important, never thought about it).

### Statistical analyses

Survey data were available from 172 Aboriginal young people. All analyses were conducted using Stata 10 Intercooled (Stata Cooperation, College Station, Texas; 2007).

The following bivariate associations were explored using χ^2 ^test and simple logistic regression: self-reported racism and each of the health outcomes (depression, overall mental health, general health); self reported racism and secondary explanatory variables (socio-demographics, life stressors, friends, school, emotions and feelings, cultural values).

All variables with a marginal statistical significance (p < 0.10) were considered for inclusion in hierarchical logistic regression models with reported racism as the primary independent variable and each of the health variables (depression, overall mental health, general health) as the outcome variable in separate models. After the first step examining relationships between reported racism and each health variable, secondary variables were entered in blocks at separate steps. Tested variables p > = 0.10 were removed at each step. Age, current level of education, and present money situation were added in the second step. Life stressors, friends, school and cultural values variables were added in the third step. The Wald test was used to assess model fit at each step of the analysis. Variance inflation factors of less than three for variables across all models indicated that multicollinearity was not present.

Interaction (i.e. effect modification) between racism and other explanatory variables in the final model was also explored (with removal set at p > = 0.10). Emotions and feelings variables were explored as potential mediators but not as secondary explanatory variables. Probit-based binary mediation with bootstrapping (5,000 replications) was utilised to produce point estimates along with bias-corrected non-parametric percentile confidence intervals[18].

## Results

Racism was reported by 52.3% of participants who had a mean age of 19.4 years (SD 3.7; range 12-26 years). Of those reporting racism, most reported a little (56.7%) or some (25.6), followed by quite a bit (12.2%) and a lot (5.6%). Participant characteristics and bivariate associations with self-reported racism are shown in Table 1. Bivariate analysis revealed a statistically significant association between self reported racism and depression (OR 2.72; 95% CI 1.46-5.10, p = 0.002), overall mental health (OR 3.31; 95% CI 1.74-6.32, p = 0.001), and general health (OR 2.53, 95% CI 1.24-5.14, p = 0.01). Participants were significantly more likely to report racism if they were aged 18-26 years, not a student and experiencing some difficulty with their present money situation. Food security, housing stability, number of friends, talking to friends with a problem, feeling safe at school, suspension or expulsion from school, feeling lonely and getting angry with racist remarks were all associated with self-reported racism at the bivariate level. Self reported racism was not associated with perceived importance of cultural values.

**Table 1:** Sample characteristics by self-reported racism (n = 172)

	% (n)	% reporting racism (n)		Unadjusted OR (95% CI)
		**Not at all**	**A little/some/quite a bit/a lot**	

**Total**		47.7 (82)	52.3 (90)	
**Socio-demographics**				
Sex				
Male	43.6 (75)	44 (33)	56 (42)	1.0
Female	56.4 (97)	50.5 (49)	49.5 (48)	0.77 (0.42-1.41)
Age (years)				
12-17	32 (55)	65.5 (36)	34.6 (19)	1.0
18-26	68 (117)	39.3 (46)	60.7 (71)	2.92 (1.50-5.70)**
Current level of education				
Primary/high school	29.1 (50)	64 (32)	36 (18)	1.0
TAFE/University	10.5 (18)	44.4 (8)	55.6 (10)	2.22 (0.74-6.64)
Not a student	60.5 (104)	40.4 (42)	59.6 (62)	2.62 (1.31-5.27)**
Present money situation				
Plenty	13.5 (23)	69.6 (16)	30.4 (7)	1.0
Get by	57.7 (98)	45.9 (45)	54.1 (53)	2.69 (1.02-7.12)**
Not enough	28.8 (49)	40.8 (20)	59.2 (29)	3.31 (1.15-9.52)**
**Life Stressors**				
Is getting enough food a problem?				
Never	67.4 (116)	57.8 (67)	42.2 (49)	1.0
Some days/often	32.6 (56)	26.8 (15)	73.2 (41)	3.74 (1.86-7.50)***
Are you looking for somewhere to live at present?				
No	55.2 (95)	54.7 (52)	45.3 (43)	1.0
Yes/unsure	44.8 (77)	39 (30)	61 (47)	1.89 (1.03-3.49)*
Worries in life				
Low	50.6 (84)	59.5 (50)	40.5 (34)	1.0
High	49.4 (82)	36.6 (30)	63.4 (52)	2.55 (1.36-4.77)**
**Friends**				
How many friends do you have?				
A lot of friends	68.8 (117)	54.7 (64)	45.3 (53)	1.0
No friends/A few	31.2 (53)	34 (18)	66 (35)	2.35 (1.2-4.61)**
Talk to friends when you have a problem				
Yes	76.7 (132)	51.5 (68)	48.5 (64)	1.0
No	23.3 (40)	35 (14)	65 (26)	1.97 (0.95-4.11)*
**School**				
Do you feel safe at school?				
Yes	52.3 (90)	56.7 (51)	43.3 (39)	1.0
Sometimes/never	47.7 (82)	37.8 (31)	62.2 (51)	2.15 (1.17-3.96)**
Have you ever been suspended or expelled from a school?				
Not at all	52.6 (90)	55.6 (50)	44.4 (40)	1.0
Once/more than once	47.4 (81)	39.5 (32)	60.5 (49)	1.91 (1.04-3.52)*
**Culture**				
Are Koori values important to you?				
Not at all	2.9 (5)	40 (2)	60 (3)	1.0
Somewhat/very important	89.5 (154)	45.5 (70)	54.6 (84)	0.8 (0.13-4.92)
Never thought about it	7.6 (13)	76.9 (10)	23.1 (3)	0.2 (0.02-1.82)
**Emotions and Feelings**				
Do you feel lonely?				
Never	31.2 (53)	69.8 (37)	30.2 (16)	1.0
Sometimes/often/always	68.8 (117)	38.5 (45)	61.5 (72)	3.7 (1.85-7.41)***
Do you get angry with racist remarks?				
No	27.9 (48)	30 (62.5)	18 (37.5)	1.0
Yes	72.1 (124)	52 (41.94)	72 (58.06)	2.31 (1.16-4.58)*
**Health Outcomes**				
Depression				
No	57 (98)	58.2 (57)	41.8 (41)	1.0
Yes	43 (74)	33.8 (25)	66.2 (49)	2.72 (1.46-5.10)**
Mental health				
Good	57.7 (97)	59.8 (58)	40.2 (39)	1.0
Poor	42.3 (71)	31 (22)	69 (49)	3.31 (1.74-6.32)***
General health				
Good/excellent	70.7 (116)	53.5 (62)	46.6 (54)	1.0
Poor/fair	29.3 (48)	31.3 (15)	68.8 (33)	2.53 (1.24-5.14)**

*p < 0.05

**p < 0.01

***p < 0.001

In the multivariable models shown in Table 2 significant relationships remained between self-reported racism and overall mental health (OR 2.67, 95% CI 1.25-5.70, p = 0.01) and between self-reported racism and general health (OR 2.17, 95% CI 1.03-4.57, p = 0.04) with the relationship between self-reported racism and depression reduced to marginal significance (OR 2.0; 95% CI 0.97-4.09, p = 0.06).

**Table 2:** Multivariable adjusted associations between reported racism and mental health, depression and general health *

	Odds ratio (95% CI) Model 1	Adjusted Odds ratio (95% CI) Model 2	Adjusted Odds ratio (95% CI) Model 3
*Depression*			
Reported racism			
Not at all	1.0	-	1.0
A little/some/quite a bit/a lot	2.72 (1.46-5.10)**	-	2.0 (0.97-4.09)
Worries in life			
Low	-	-	1.0
High	-	-	3.82 (1.84-7.95)***
Is getting enough food a problem?			
Never	-	-	1.0
Some days/often	-	-	2.32 (1.06-5.08)*
R^2#^	0.071	-	0.258
N	172	-	166

*Mental Health*			
Reported racism			
Not at all	1.0	1.0	1.0
A little/some/quite a bit/a lot	3.31 (1.74-6.32)***	2.99 (1.53-5.84)***	2.67 (1.25-5.70)**
Present money situation			
Plenty	-	1.0	-
Get by	-	1.80 (0.59-5.46)	-
Not enough	-	4.36 (1.33-14.22)*	-
Worries in life			
Low	-	-	1.0
High	-	-	4.78 (2.23-10.24)***
How many friends do you have?			
A lot of friends	-	-	1.0
No friends/A few	-	-	2.50 (1.11-5.64)*
Are you looking for somewhere to live at present?			
No	-	-	1.0
Yes/unsure	-	-	2.85 (1.34-6.06)**
R^2#^	0.099	0.160	0.383
N	168	167	161

*General Health*			
Reported racism			
Not at all	1.0	1.0	1.0
A little/some/quite a bit/a lot	2.53 (1.24-5.14)**	2.49 (1.18-5.24)*	2.17 (1.03-4.57)*
Present money situation			
Plenty	-	1.0	-
Get by	-	1.89 (0.5-7.1)	-
Not enough	-	4.09 (1.04-16.14)*	-
Are you looking for somewhere to live at present?			
No	-	-	1.0
Yes/unsure	-	-	3.93 (1.88-8.22)***
R^2#^	0.061	0.129	0.78
N	164	162	164

#McKelvey-Zavoina pseudo R^2 see ^[42]

*Models shown exclude tested explanatory predictors with significance level p > = 0.10

Total number of worries and number of friends were both found to be effect modifiers for the association between self-reported racism and overall mental health in the final model. After controlling for number of friends and housing stability, the association between reported racism and overall mental health was stronger for those with fewer worries (OR 5.7, 95% CI 1.67-19.43; p = 0.005) than for those with more worries where the association was not significant (OR 1.45; 0.50-4.17; p = 0.49 After controlling for worries and housing stability, the association between reported racism and overall mental health was stronger for those with no or few friends (OR 11.39; 95% CI 2.29-56.69; p = 0.003) than for those with lots of friends where it was not significant (OR 1.60; 95% CI 0.65-3.90; p = 0.305). No statistically significant interaction terms were found for multivariate models with either depression or general health as the outcome.

Getting angry at racist remarks was found to mediate the relationship between self-reported racism and general health (β coefficient = 0.05, Bias-corrected CI 0.004-0.16, 22% of effect mediated). No significant mediators were found between self-reported racism and either overall mental health or depression.

## Discussion

This study examined the health effects of racism among Aboriginal young people aged 12-26 years living in Melbourne, Victoria. A large proportion (52.3%) of all participants reported they had experienced racism. This is a considerably higher prevalence of racism than reported amongst 12-17 year old Aboriginal young people in the WAACHS (22%). Although the WAACHS did not find a significant difference in prevalence of self reported racism across levels of remoteness, this finding may reflect higher prevalence of racism within an urban specific survey compared to a statewide survey. Possibly the finding may in part be explained by differences between the proportion of Aboriginal people within each of state. Victoria is the state with the smallest proportion of Aboriginal people who are 0.6% of the total population compared to Western Australia where Aboriginal people are 3.8% of the total population, second only to the Northern Territory [22]. This explanation is supported by suggestions that being an invisible minority exposes urban Aboriginal Australians to increased risk of discrimination [23,29-32].

It has also been found internationally that ethnic minorities who live in areas of high ethnic density report fewer experiences of racism compared to their peers living in areas of low ethnic density [33].

The higher prevalence of racism in this study may also reflect the age range of participants. Sub-group analysis by age indicates that older participants aged 18-26 years were more likely to report racism (60.7%) than the younger 12-17 year olds (34.6%) although the prevalence in this younger group is still higher than that in the comparable sample from the WAACHS. Possibly the increased reporting of racism with age found in this study reflects suggestions from research involving African Americans that adolescence and young adulthood is a time of increased recognition of minority status and awareness of discrimination brought about by increased function outside of family contexts and development of more complex cognitive and moral reasoning skills [13,34].

Finally, another likely explanation for this higher reporting of racism is the measurement item used. While both studies measured self reported racism using a single item, the YPP item was not time bound thus considered a life-time history of racism whereas the WAACHS limited experiences to the past six months. This reflects the puzzling picture more broadly in Australia where prevalence of reported racism amongst Aboriginal and Torres Strait Islander adults ranges between 16-93% and varies considerably across studies conducted in different geographical locations (including locally based and national surveys), using different timeframes (e.g. 4 weeks, 6 months, 12 months, ever) within both single and multi-item measures [19-21,35,36]. More research is needed to examine the most appropriate items for measuring self-reported racism and to determine the prevalence of perceived racism for Aboriginal and Torres Strait Islander people of all ages and across geographical locations.

This study also found that self-reported racism was significantly associated with poor overall mental health and with poor general health, and marginally associated with depression, in the multivariate analysis after controlling for confounders. The effect of racism on poor mental health was found to be stronger for those with no or few friends compared to those with many friends. This suggests that availability of social support may protect against the negative impact of racism on mental health. A recent meta-analysis of perceived discrimination and health examined studies exploring the interactive effect of social support on the relationship between perceived discrimination and mental health and found social support either lessened or had null effect on this relationship [11]. The role of social support as a potential buffer against the negative effects of racism as well as the effectiveness of social support as a strategy for coping with racism require further elucidation [37].

This study also found the effect of racism on mental health to be stronger amongst those with fewer worries compared to those with more worries. This somewhat anomalous finding contradicts other studies as well as the wider stress literature which suggests that stressors combine in additive and interactive ways [2,38]. Further exploration of the interactions between experiences of racism and other stressors may shed light on this finding.

A novel finding of this study was that getting angry at racist events was a mediator of the relationship between self-reported racism and general health. This finding is particularly significant given that anger has been identified as the most common response to experiences of racism for Aboriginal and Torres Strait Islander Australians [5,21]. That racism evokes anger is also well recognised internationally, where attention is now increasingly placed on the need to consider anger as an important coping response to racism, including both outward anger expression and internal anger suppression [37]. Few studies have directly examined the effects of anger coping on health outcomes with more work needed to examine the health effects on individuals of using confrontation in the face of race-related conflict, as well as how to manage the emotional burden created by the anger [37]. Studies that do exist have found African American adults consistently report negative effects of anger suppression on both blood pressure and psychological distress, likely due to rumination if issues are not settled appropriately [37]. However, studies have also reported that while anger expression may lead to less rumination and greater feelings of self-efficacy, it may also threaten social relationships and lead to anxiety about retaliation or abandonment [37].

Findings of this study should be considered in the context of several limitations. The sample is not representative of the Australian Aboriginal population. Although 32 longitudinal studies suggest that the primary direction of causation is from racism to ill-health, [2,10,11] the cross sectional design limits conclusions about causal directions in this study. Measures of mental health and depression used in this study have not been subject to rigorous psychometric testing to establish their validity amongst urban Aboriginal young people. However, there is a lack of such robust tools for this population, and the findings of this study are consistent with those amongst other population groups. Failure to identify any significant moderators may have been due to the low power of moderation tests [39] combined with the small sample size in this study. Similarly, the small sample size contributed to the large confidence intervals around significant point estimates in this study and may have precluded identification of weaker mediation effects. The use of a single item to measure experiences of racism rather than a multi-item measure is unlikely to capture all experiences of racism and thus racism may be underestimated in this study [10,40]. Also, it is important to note this single item does not include a timeframe and so can be considered a measure of lifetime exposure. While it is considered important, and is most common, to consider cumulative exposure to racism over the lifetime [41], issues of unreliable recall and recall bias affecting the reliability and validity of self-report measures are considered more problematic when the recall timeframe of stressors is longer and when data is collected retrospectively [2]. Measures that examine multiple domains of racism across multiple settings and in a range of contexts are thus recommended [2]. As it is possible that some of the differences in self-reported racism related to age found in this study are associated with the measures used and that questions have different meanings for those of different ages, such robust measures that are appropriate and relevant to children and young people are also required for future studies in this field.

## Conclusion

The findings of this study highlight the need to acknowledge and address racism as an important determinant of health and wellbeing for Aboriginal young people in Australia. Further research is required using multiple item and multiple dimension measures of racism to better understand how these events are experienced by young people, including specifically with Aboriginal young people in urban areas.

## Competing interests

The authors declare that they have no competing interests.

## Authors' contributions

NP conducted the data analysis and wrote the manuscript. YP assisted with data analysis and writing the manuscript. PS was involved in the design and coordination of the project, participated in data collection and edited the manuscript. JL assisted with data analysis and edited the manuscript. All authors have read and approved the final manuscript.

## Pre-publication history

The pre-publication history for this paper can be accessed here:

http://www.biomedcentral.com/1471-2458/11/568/prepub
